# Genomic Analysis of Two Representative Strains of *Shewanella putrefaciens* Isolated from Bigeye Tuna: Biofilm and Spoilage-Associated Behavior

**DOI:** 10.3390/foods11091261

**Published:** 2022-04-27

**Authors:** Zhengkai Yi, Jing Xie

**Affiliations:** 1 College of Food Science & Technology, Shanghai Ocean University, Shanghai 201306, China; d200300064@st.shou.edu.cn; 2 Shanghai Professional Technology Service Platform on Cold Chain Equipment Performance and Energy Saving Evaluation, Shanghai 201306, China; 3 National Experimental Teaching Demonstration Center for Food Science and Engineering, Shanghai 201306, China; 4 Shanghai Engineering Research Center of Aquatic Product Processing & Preservation, Shanghai 201306, China

**Keywords:** *Shewanella putrefaciens*, complete genome sequence, spoilage, biofilm, extracellular protease

## Abstract

*Shewanella putrefaciens* can cause the spoilage of seafood and shorten its shelf life. In this study, both strains of *S. putrefaciens* (YZ08 and YZ-J) isolated from spoiled bigeye tuna were subjected to in-depth phenotypic and genotypic characterization to better understand their roles in seafood spoilage. The complete genome sequences of strains YZ08 and YZ-J were reported. Unique genes of the two *S. putrefaciens* strains were identified by pan-genomic analysis. In vitro experiments revealed that YZ08 and YZ-J could adapt to various environmental stresses, including cold-shock temperature, pH, NaCl, and nutrient stresses. YZ08 was better at adapting to NaCl stress, and its genome possessed more NaCl stress-related genes compared with the YZ-J strain. YZ-J was a higher biofilm and exopolysaccharide producer than YZ08 at 4 and 30 °C, while YZ08 showed greater motility and enhanced capacity for biogenic amine metabolism, trimethylamine metabolism, and sulfur metabolism compared with YZ-J at both temperatures. That YZ08 produced low biofilm and exopolysaccharide contents and displayed high motility may be associated with the presence of more a greater number of genes encoding chemotaxis-related proteins (*che*X) and low expression of the *bpf*A operon. This study provided novel molecular targets for the development of new antiseptic antisepsis strategies.

## 1. Introduction

Aquatic products are regarded as an important food source globally owing to its low-fat content and rich animal protein. However, aquatic products are highly perishable foods after death, even under refrigerated conditions. Microorganisms are essential in the spoilage of aquatic products, even with the rapid development of modern preservation technologies [1,2]. The main microorganisms responsible for food spoilage are known as specific spoilage organisms (SSOs) [3]. These SSOs can break down nitrogenous compounds (amino acids and proteins) in aquatic products into ammonia, biogenic amines, sulfides, and volatile compounds (including aldehydes, ketones, alcohols, and organic acids), leading to the degradation of sensory properties and making fish products unacceptable [4,5].

Although low temperatures inhibit the growth and metabolism of most microbes, many studies showed that *S. putrefaciens* can reduce the shelf life of refrigerated seafood, such as tuna [6], Pacific white shrimp [7], and cod [8]. Most *S. putrefaciens* strains can reduce trimethylamine oxide (TMAO) to trimethylamine (TMA) [9] and decarboxylate specific amino acids to biogenic amines, including putrescine, histamine, and cadaverine [10]. *S. putrefaciens* can also form biofilms in the aquatic matrix to enhance its adsorption capacity [11], degrade myofibrillar proteins in fish meat, and oxidize lipids [12]. However, relatively few studies have focused on the genome of *S. putrefaciens*, when investigating its relationship with its spoilage potential.

In recent years, genome-wide mining has contributed to the understanding of spoilage-associated metabolic pathways in SSO [13]. Exploring spoilage-related metabolic pathways by gene mining is essential for gaining insight into the spoilage behavior of spoilage bacteria. Chen et al. [14] reported the genome-wide sequence of *S. putrefaciens* WS13. However, the spoilage potential of *S. putrefaciens* has not been resolved at the genomic level. In addition, *S. putrefaciens* possesses good cold adaptability under low temperature conditions, which may be related to the expression of genes regulating fatty acid metabolism [15]. To date, there are no studies reporting the association between spoilage-related genes and phenotypic traits of *S. putrefaciens*.

The aim of this work was to reveal the mechanisms of *S. putrefaciens* underlying the spoilage activity at the genetic level. To achieve this, the whole genome sequences of two *S. putrefaciens* strains with different spoilage abilities were studied and compared to reveal the spoilage-associated genetic differences and identify key spoilage-causing genes. Phenotypes and related genotypes, including growth under stress, biofilm formation, motility, protein hydrolysis, lipolytic activity, TMA, and hydrogen sulfide (H_2_S) production capacity, were investigated in both strains to elucidate the relationship between spoilage-related genes and bacterial phenotypes. This study is helpful for the search for new spoilage factors of *S putrefaciens* and can be further verified by molecular biology methods. Our results provide new directions in the advancement of spoilage detection and prevention methods and identify novel involved in microbe-mediated fish spoilage.

## 2. Materials and Methods

### 2.1. S. putrefaciens Strains and Cultures

*S. putrefaciens* YZ08 and YZ-J strains were isolated from spoiled bigeye tuna during 4 °C storage for 8 days. Spoiled bigeye tuna was determined by sensory evaluation as described by Yi and Xie [16]. A total of 25 g of spoiled tuna flesh was homogenized in 225 mL of 0.85% sterile saline and serial dilutions were prepared. For bacterial purification, all the isolates from the highest dilution plate (nutrient broth, containing 30–100 isolates) were incubated in nutrient broth for 48 h at 30 °C and were subsequently purified on iron agar (IA) at 30 °C. *Shewanella* spp. is an H_2_S-producing bacterium that produces black clones on IA. The H_2_S-producing bacteria strains were dominant species (20/50). All purified colonies were identified by 16S rRNA gene sequencing using primers 27F (5′-AGAGTTTGATCMTGGCTCAG-3′) and 1492R (5′-GGTTACCTTGTTACGACTT-3′). In a preliminary experiment, 14 *S. putrefaciens* strains were inoculated into sterilized tuna meat. The sterile fish blocks were prepared according to Li et al. [12]. After inoculation, fish blocks were placed in sterile bags for refrigeration at 4 °C for 8 days. Through preliminary sensory identification, the clones associated with the best and the worst quality of inoculated fish meat were selected for further study (identified as *S. putrefaciens* YZ08 and YZ-J). Purified strains were stored in tryptose soya broth (TSB) contained 25% glycerine at −80 °C. Before use, stains were pre-cultured in TSB for 18 h and then cultured in TSB at 30 °C for 8 h. Bacterial cell cultures at the logarithmic phase (8 log CFU/mL, OD_600_ ≈ 0.8) were used for inoculation in subsequent experiments (including bacterial DNA extraction, growth, biofilm formation, motility assays, protein hydrolysis, lipolysis, measurement of spoilage-related indicators and RT-qPCR).

### 2.2. Complete Genome Sequencing of S. putrefaciens YZ08 and YZ-J and Functional Annotation

The high-quality genomic DNA was extracted from two *S. putrefaciens* strains with a Bacterial DNA Kit (OMEGA). Genome sequencing was performed by Shanghai Biozeron Technology Co. Ltd. (Shanghai, China) using Illumina NovaSeq 6000 sequencing platform and PacBio RSII platform. A total of 3μg of genomic DNA (150 ng/μL) were used for sequencing library construction. Two libraries with insert sizes of approximately 400 bp and 15–20 kb was constructed and sequenced on an Illumina NovaSeq 6000 sequencing platform and a PacBio RSII platform, respectively. Raw data obtained from the Illumina NovaSeq 6000 sequencing platform and PacBio RSII platform were quality controlled (remove low-quality reads and repeat reads) for further assembly. First, Illumina sequencing data were assembled using SOAPdenova v1.05 and then were compared with the PacBio sequencing data corrected errors. Next, the corrected data were assembled using Celera Assembler v8.0 to generate the scaffolds. Finally, the assembled scaffolds were mapped back to Illumina clean reads using GapCloser v1.12 for gap closing.

Functional annotation was performed using databases of non-redundant (NR) protein, Kyoto Encyclopedia of Genes and Genomes (KEGG), Swiss-Prot, Cluster of Orthologous Groups (COG), and Gene Ontology (GO). Genomic tRNAs and rRNAs were analyzed using tRNAscan-SE v1.3.1 (University of California, California, USA) and RNAmmer v1.2 (Technical University of Denmark, Lyngby, Denmark). Secretory proteins were predicted using the signal peptide prediction tool signalP v5.0 (Technical University of Denmark, Lyngby, Denmark) [17].

For the identification of genes associated with spoilage behavior, key proteins related to spoilage metabolism were collected. Candidate genes were obtained by searching all predicted proteins in the YZ08 and YZ-J genomes (E-value = 1E10; coverage ≥ 70%; identity ≥ 35%) using the blastP algorithm. Candidate genes were further confirmed by protein, COG, and GO functional annotation [18]. In addition, relevant metabolic genes were obtained directly by KEGG pathway analysis.

### 2.3. Pan-Genome Analysis and Genome Mining of S. putrefaciens YZ08 and YZ-J

Pairwise genome alignment and visualization analysis of *S. ptrefaciens* YZ08 and YZ-J strains were performed using MAUVE v2.4.0 (University of Wisconsin, Wisconsin, USA) [19]. All 10 *S. putrefaciens* complete genomes in NCBI were subjected to pan/core genome analysis using the Bacterial Pan Genome Analysis tool (BPGA v1.3) [20]. The NCBI accession numbers for 8 of the 10 strains (excluding strains YZ08 and YZ-J) were NZ_CP066370, NZ_CP066369, NZ_CP046329, LR134303, CP015194, NZ_CP078038, NZ_CP028435, and CP070865. Each nucleotide sequence was analyzed using default settings. In BPGA, homologous protein clusters were identified using USEARCH (a clustering tool) with a threshold of 0.5 and a phylogenetic tree was constructed based on the core genome. In addition, pan-genomic analysis was performed for strains YZ08 and YZ-J and unique genes were subjected to COG and KEGG functional annotation.

### 2.4. Growth and Biofilm Formation of S. putrefaciens YZ08 and YZ-J under Stress Conditions

*S. putrefaciens* strains YZ08 and YZ-J were pre-cultured in Luria Broth (LB) medium at 30 °C for 12 h. As a control, each strain was inoculated into LB medium (pH 7.0) at a ratio of 1:1000 and incubated at 30 °C. To induce pH stress, the incubation temperature was kept constant (30 °C), but the pH of the LB medium was regulated to 6.0 using HCl; for NaCl stress, the NaCl concentration was adjusted to 5% by adding NaCl; for nutrient stress, the medium was diluted to 20% by adding distilled water; for temperature stress, the temperature was set at 4 °C. The bacteria were incubated under stress conditions for 168 h, and the total cell numbers was measured every 12 h. The total cell numbers were performed using plate count agar (PCA). Samples (1 mL) were serially diluted 10-fold with normal saline, and dilutions (0.1 mL) were spread on PCA. Plates were placed at 30 °C for 48 h and the total cell numbers were determined.

One milliliter of the dilutions (1:1000) of the above pre-cultures under various stress conditions were transferred to a 48-well plate. The plates were incubated under static conditions for 12, 24, 48, and 72 h. Biofilm formation were determinate by Yan and Xie [11]. Briefly, after culture, the supernatant was carefully discarded and the adherent cells were washed twice using saline (0.85%) and dried, then stained with 0.2% crystal violet for 15 min, washed and dried. Finally, it was dissolved in 95% ethanol to determine OD_600_. The total cell numbers in LB medium were also determined for normalization. The total cell numbers were determined by serial dilution as described in Section 2.4. The normalized results were expressed as the ratio of the OD_600_ to the total number of cells.

### 2.5. Motility Assays

Swarming and swimming motility were measured on LB cultures containing 1.5% and 0.3% agar, respectively. Five microliters of bacterial culture (1 × 10^8^ CFU/mL) was dropped onto agar plates and incubated at 4 °C and 30 °C for 24, 48, and 72 h, and the diameters of the motility zones were measured, respectively. 

### 2.6. Proteolytic and Lipolytic Activity

Proteolytic activity was measured on agar plates containing 5% skimmed milk (prepared with deionized water containing 5% skimmed milk powder and 2% agar), and lipolytic activity was measured on triglyceride agar (Solarbio, Beijing, China). Incubation was performed at 30 °C for 24, 48, and 72 h and 4 °C for 1, 2, and 4 days, respectively. Finally, proteolytic and lipolytic activity was determined according to the size of the produced halos.

### 2.7. Spoilage-Related Indicators

#### 2.7.1. Determination of TMA Content

Both strains of *S. putrefaciens* were inoculated in LB medium with TMAO (10 mM) and phosphate buffer saline (PBS, 100 mM) for 6 days at 4 °C and 48 h at 30 °C. TMA content was determined using the picric acid method [21].

#### 2.7.2. Determination of Biogenic Amines (BAs) Content

Both strains of *S. putrefaciens* were inoculated in LB medium with 0.5% L-ornithine monohydrochloride, L-arginine monohydrochloride, and lysine monohydrochloride with 0.005% pyridoxal-5′-phosphate for 6 days at 4 °C and 48 h at 30 °C. BAs were extracted according to Zhuang et al. [22], then separated and quantified using HPLC (SHIMADZU, LC-2010C HT, Kyoto, Japan) and COSMOSIL 5C18-PAQ columns after derivatization with dansyl chloride. Chromatographic conditions were according to Hong et al. [23].

#### 2.7.3. Preparation and Inoculation of Sterile Tuna Juice

Minced bigeye tuna back muscle (2 kg) was homogenized and boiled for 5 min with 2 L of distilled water. After adding 1.6 g/L TMAO, 40 mg/L L-cysteine and L-methionine, the filtrate was sterilized (121 °C, 15 min), yielding sterile bigeye tuna juice. The *S. putrefaciens* strains were separately inoculated into the fish juice at a final concentration of approximately 5 log CFU/mL and stored at 4 and 30 °C.

#### 2.7.4. Determination of H_2_S Content

The H_2_S content in fish juice was determined using a H_2_S concentration determination kit (Beijing Solabao, Beijing, China) and was expressed as μmol/mL.

#### 2.7.5. Determination of Exopolysaccharide Content and Extracellular Protease Activity

The exopolysaccharide of the inoculated fish juice was extracted according to the method of Feng et al. [24]. Nine milliliters of the inoculated fish juice was transferred to a six-well plate and incubated at 30 °C for 24, 48, and 72 h and at 4 °C for 1, 2, and 4 days, respectively. After carefully removing the cultures from the wells, the plate was cleaned 3 times with PBS to remove residual bacterial cells, 9 mL of PBS was added to each well, followed by sonication at 50 kHz for 5 min to dissolve the exopolysaccharides that had adhered to the walls of the wells (sonication can lyse cells in biofilms, resulting in the release of intracellular content). After centrifugation at 12,000× *g* for 15 min at 4 °C, the obtained supernatant was used to measure exopolysaccharide content using the phenol-sulfuric acid method [25], with glucose serving as a standard. Exopolysaccharide content was expressed as μg/mL.

Extracellular protease activity was determined according to Anbu [26], with some modifications. After centrifugation at 10,000× *g* at 4 °C, the supernatant (500 μL) of the fish juice was added to an equal volume of 1% (*w*/*v*) casein substrate solution and incubated at 37 °C for 10 min. The reaction was terminated by adding 1 mL of TCA (20%). Finally, after centrifugation (12,000× *g*, 10 min), the tyrosine content was determined using the Folin method [27]. The results were expressed as U/mL.

### 2.8. RT-qPCR

*S. putrefaciens* strains YZ08 and YZ-J were inoculated in LB medium at 30 °C until the log phase (OD_600_ = 0.8). Total RNA was isolated from cultured cells using a Spin Column Bacteria Total RNA Purification Kit (Sangon, Shanghai, China) and reverse-transcribed into cDNA using an AMV First Strand cDNA Synthesis Kit (Sangon, Shanghai, China) with random primer p(dN)_6_. qPCR was performed using SG Fast qPCR Master Mix (SYBR Green) (Sangon, Shanghai, China). The sequences of the primers used for qPCR are listed in Table S1. mRNA levels were normalized to that of the 16 S rRNA gene. The relative expression levels of each gene were determined using the 2^−ΔΔCt^ method [28].

### 2.9. Statistical Analysis

All data were analyzed using SPSS 19.0 (IBM, Chicago, IL, USA). The Student’s *t*-test was employed for comparisons between two groups. One-way analysis of variance (ANOVA) with Duncan’s post-hoc test was used for multiple groups. *p* < 0.05 were considered significant. All experiments were repeated at least three times.

## 3. Results and Discussion

### 3.1. Identification and Genome Properties of S. putrefaciens Strains YZ08 and YZ-J

**Figure 1:**
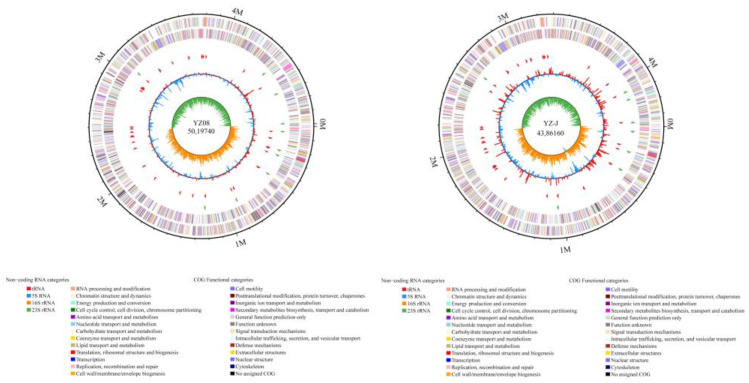
Map of the circular genome of *Shewanella putrefaciens* YZ08 and YZ-J. Genome size (first circle); coding DNA sequences on forward and reverse chains based on COGs categories (second and third circles); forward strand and reverse strand non-coding RNA (ncRNA) (fourth and fifth circle); guanine-cytosine (GC) content (sixth circle) and GC skew (fifth circle).

The complete genome sequences were submitted in GenBank with accession numbers CP080633 (for YZ08) and CP080635 (for YZ-J). The genomes of *S. putrefaciens* YZ08 and YZ-J were determined to be 5,019,740 bp and 4,386,160 bp long, with average GC contents of 47.69% and 46.53%, respectively (Figure 1). Genes were annotated in multiple databases: NR, 4353 and 3806; Swiss-Prot, 3122 and 2842; COG, 3803 and 3464; KEGG, 2314 and 2194; GO, 1456 and 1429; VFDB, 527 and 535; and CAZy, 80 and 64. The genome contained 4443 and 3890 of genes in which 4301 and 3761 (96.80% and 96.68%) were assigned predicted proteins in YZ08 and YZ-J, respectively.

### 3.2. Gene Function Analysis

#### 3.2.1. COG, GO, and KEGG Function Classification Analysis of the Two Strains

**Figure 2:**
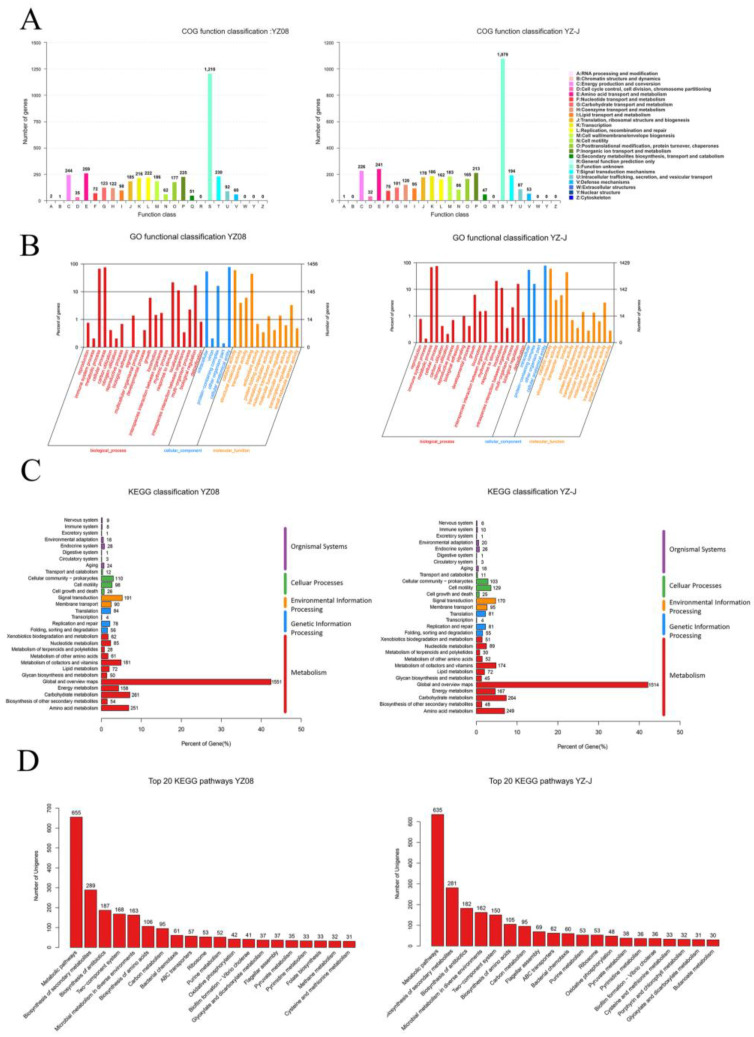
Whole genome sequence analysis of *S. putrefaciens* YZ08 and YZ-J. (**A**), Cluster of orthologous groups (COG). (**B**), Gene ontology (GO). (**C**), Kyoto Encyclopedia of Genes and Genomes (KEGG) Classification. (**D**), Top 20 KEGG pathways.

The unknown function category contained the largest number of genes for both *S. putrefaciens* strains. Both strains showed similar trends by which amino acid transport and metabolism, and energy production and conversion were the main COGs (Figure 2A). For energy production and conversion, the four most abundant COGs were COG0243, COG1012, COG0437, and COG1301 for YZ08, and COG1012, COG1053, COG0243, and COG0437 for YZ-J. COG0437 plays a crucial role in various electron transfer processes and several enzymatic reactions [29]. In response to environmental stresses, two Na+ ions were transported by COG1301 [30]. A similar COG functional classification was also found in *S. baltica* 128 (accession number CP028730). The differences in COG classification may be indicative of differences in metabolism and adaptability to the environment between the two strains. *S. putrefaciens* YZ08 and YZ-J genes were assigned to three function classifications by GO analysis (Figure 2B). The genes of the YZ08 and YZ-J strains in the biological process category were divided into 20 subfunctions, most of which were associated with cellular processes (GO:0009987), metabolic processes (GO:0008152), and response to stimulus (GO:0050896), which was consistent with the features reported for *Shewanella* spp. [13]. A similar GO functional classification was found for *S. putrefaciens* XY07 (accession number CP070865). The results for the KEGG pathway analysis of *S. putrefaciens* YZ08 and YZ-J genes are shown in Figure 2C,D. In the five KEGG pathways of the two strains, metabolism comprised the largest number of genes, followed by environmental information processing, cellular processes, and genetic information processing. Some pathways may be related to the distinct phenotypes shown by the two strains, such as two-component system (ko02020), microbial metabolism in diverse environments (ko01120), biosynthesis of amino acids (ko01230), bacterial chemotaxis (ko02030), ABC transporters (ko02010), biofilm formation-Vibrio cholerae (ko05111), flagellar assembly (ko02040), and cysteine and methionine metabolism (ko00270). Two-component systems (TCS) can translate extracellular signals into gene expression patterns that facilitate bacterial regulation of various physiological functions. ABC transporters (ko02010) are transmembrane proteins that use energy to transport substrates into the cell [31].

#### 3.2.2. Genome Synteny and Pan-Genome Analysis

**Figure 3:**
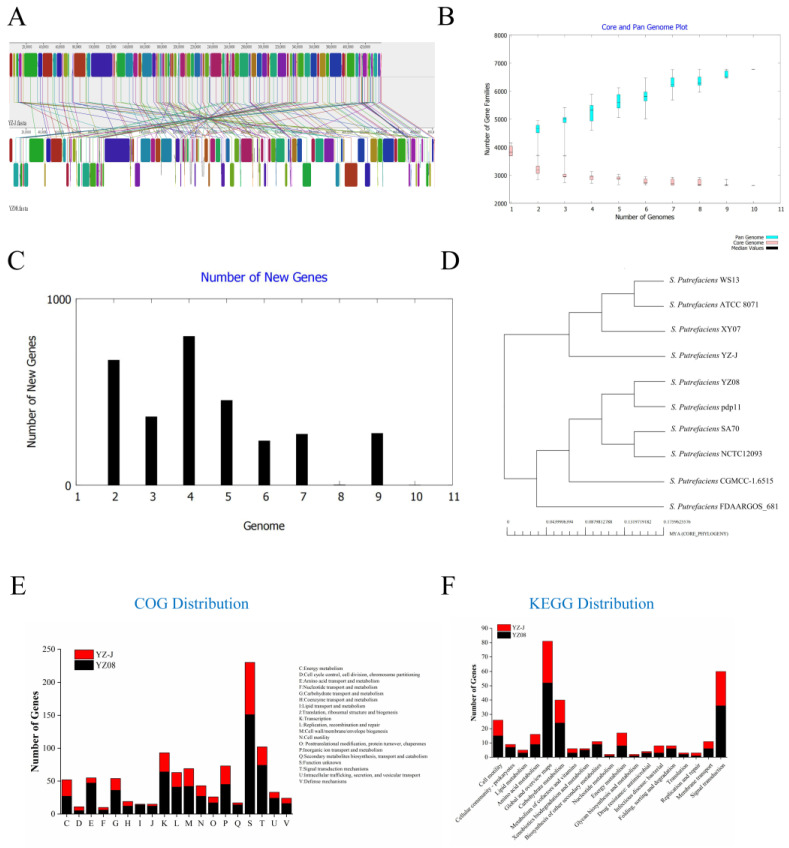
Genome synteny and pan-genome analysis of *S.putrefaciens*. (**A**) The mauve visualization of the whole genomes of *S. putrefaciens* YZ08 and YZ-J. The homologs distributed in these genomes are connected by lines; (**B**) pan-genome and core genome profile of different 10 *S. putrefaciens* strains; (**C**) prediction of increase in gene number when adding new genome; (**D**) phylogenetic tree based on pan genomes; (**E**) COG and (**F**) KEGG distribution of unique genes based on pan-genome of *S. putrefaciens* YZ08 and YZ-J.

To explore the genetic differences between both strains, a genome synteny and pan-genome analysis were also investigated. As shown in Figure 3A, analysis of the genome sequences using MAUVE revealed that *S. putrefaciens*YZ08 and YZ-J shared many homologous regions. Although large genomic rearrangements and inversions were found in both strains, overall, the YZ08 and YZ-J genomes shared a large number of homologous regions.

The pan-genome of the 10 *S. putrefaciens* strains included 2637 core genes, 10,413 accessory genes and 1944 unique genes. Analysis of the pan-genome and core genome maps of oral *S. putrefaciens* (Figure 3B,C) showed that the number of the pan-genome increased, while that of the core genome decreased, indicating that *S. putrefaciens* has an open pan-genome and has the ability to survive in a variety of environments [13]. To investigate the phylogenetic relationships among the 10 strains, a phylogenetic tree was generated based on 2637 core genes (Figure 3D). *S. putrefaciens* YZ08 showed the greatest genetic relationship with the pap11 strain, while YZ-J was close to XY07, ATCC8071, and WS13.

The pan-genome of *S. putrefaciens* YZ08 and YZ-J was investigated. The unique genes of both strains were listed in Tables S2 and S3. The unique genes of the two strains were annotated using KEGG and COG distribution (Figure 3E,F). In COG distribution, many unique genes of both strains were related to signal transduction mechanisms, which were involved in the regulation of various life activities of microorganisms. Key biofilm regulatory genes were identified in strain YZ08, annotated as COG5001 (diguanylate cyclase phosphodiesterase) in COG and classified as ko05111 (biofilm formation—Vibrio cholerae) in KEGG. These genes may play an inhibitory role in biofilm formation [32]. YZ08 may possess stronger amino acid metabolic activity than YZ-J owing to a greater abundance of COG0834 components (ABC-type amino acid transport/signal transduction system). KEGG distribution showed that only YZ08 was involved in trimethylamine metabolism in methane metabolism (ko00680). Both strains contained genes coding for proteins with functions in membrane transport (ABC transporters). These transporters are involved in transporting a large number of endogenous substrates and exogenous compounds across lipid membranes and are associated with many important biological processes, such as the release of secreted proteins, cellular detoxification, lipid homeostasis, ion channel regulation, and ribosome assembly [33]. In addition, COG and KEGG distributions indicated that YZ08 possessed more cell motility-related genes than YZ-J, especially those associated with bacterial chemotaxis. In conclusion, the pan-genomic analysis provided new insights into the differential genetic content of the two strains.

### 3.3. Stress Adaptation

**Figure 4:**
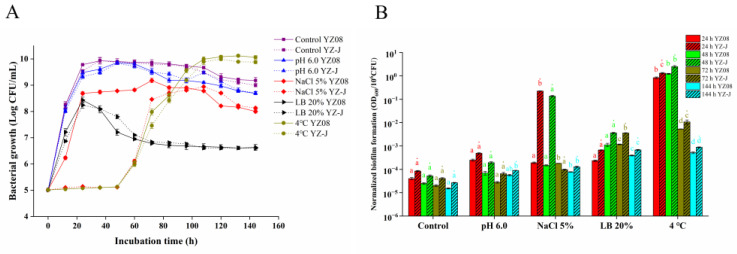
Growth curves (**A**) and biofilm formation (**B**) for *Shewanella putrefaciens* YZ08 and YZ-J under different stress conditions. The significance analysis of the same strain under different stress conditions at the same culture time was conducted; different lowercase letters indicate a significant difference (*p* < 0.05, the different styles of letters are used to distinguish the different groups).

During food processing, microbes are subjected to a range of stresses, such as temperature, salt, pH, and nutrition stresses. The growth and biofilm formation of *S. putrefaciens* YZ08 and YZ-J under different stress conditions are shown in Figure 4. The growth of both YZ08 and YZ-J in 5% NaCl and 20% LB was reduced compared with that in the control group (Figure 4A). The lag period for the growth of the two strains in the 4 °C groups was substantially longer than that of the control, but the maximum cell concentrations of the two strains were similar, which was consistent with the previous study [15]. The growth rate of YZ08 was higher than that of YZ-J in 5% NaCl; however, no significant differences were observed between the two strains for the other stress conditions. The Normalized biofilm formation rates of two strains in the 5% NaCl, 20% LB and 4 °C groups were higher than those in the control group, reaching significance in the 4 °C groups for both strains (*p* < 0.05) (Figure 4B). Low temperatures promote the expression of related genes to enhance the formation of biofilms [11]. Similarly, the higher normalized biofilm formation rates recorded under pH 6.0, 5% NaCl, and 20% LB stress relative to the control condition may be related to the formation of a greater amount of biofilm to protect the bacteria under stressful conditions [13]. In addition, the normalized biofilm decreased from 24 h to 144 h in the control, pH 6.0, NaCl 5%, and 4 °C groups, likely because the biofilm at this stage was in the dispersal period [34]. In all groups, the normalized biofilm formation of YZ08 was lower than that of YZ-J.

**Figure 5:**
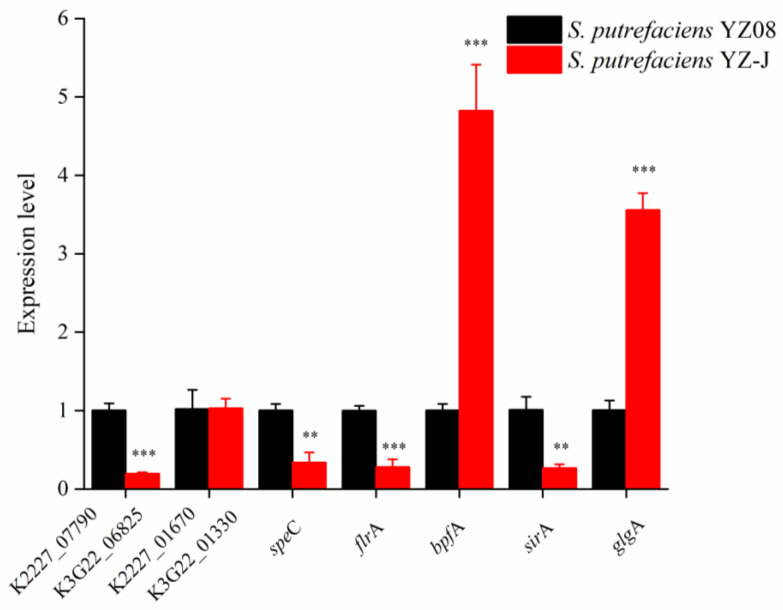
Comparison of the expression levels of biofilm formation-related and spoilage-related genes associated with salt stress between *Shewanella putrefaciens* strains YZ-J and YZ08. Gene expression levels in *S. putrefaciens* YZ-J are expressed as values relative to the control group (*S. putrefaciens* YZ08). ** *p* < 0.01, *** *p* < 0.001.

A series of stress-related genes of *S. putrefaciens* YZ08 and YZ-J, including temperature, pH, NaCl, and nutrient stresses, is shown in Table 1. The cold shock genes *csp*A and *csp*D in *L. monocytogenes* are required to induce its growth at low temperatures [35,36] and may exert a similar function in *S. putrefaciens* YZ08 and YZ-J. Three *csp*A/*csp*D genes (K2227_07410, K2227_08825, K2227_12570, and K3G22_06460, K3G22_07485, K3G22_10845, respectively) were identified in the YZ08 and YZ-J genomes, which may explain the similar cold adaptability of the two strains. Furthermore, the genomes of both YZ08 and YZ-J contained eight genes encoding stress-related F0F1 ATP synthase, which is associated with the synthesis of ATP using ion translocation [37]. Interestingly, YZ08 contained six genes encoding sodium: proton antiporter and one encoding a transporter protein (K2227_13575) related to osmotic pressure, whereas YZ-J contained only four genes encoding sodium: proton transporters and none coding for the osmotic pressure-related transporter protein. This observation may partially explain the better growth of the YZ08 strain under 5% NaCl relative to that of strain YZ-J. The presence of genes encoding choline/glycine/proline betaine transporter and plasma membrane protein involved in salt tolerance indicated that *S. putrefaciens* maintains osmotic balance using a compatible solutes strategy when exposed to osmotic stress. RT-qPCR was used to study the expression of osmotic stress-related genes (encoding choline/glycine/proline betaine transporter and plasma membrane protein—K2227_07790 and K2227_01670, respectively, in YZ08 and K3G22_06825 and K3G22_01330, respectively, in YZ-J). As shown in Figure 5, the expression of the genes encoding choline/glycine/proline betaine transporter were significantly higher in strain YZ08 than in strain YZ-J (*p* < 0.001), which was consistent with YZ08 being better adapted to a high salt environment relative to YZ-J. However, the expression levels of the gene encoding the plasma membrane protein did not show significant differences between in the two strains (*p* > 0.05). In addition, YZ08 and YZ-J shared similar genes encoding amino acid synthases.

### 3.4. Motility

**Figure 6:**
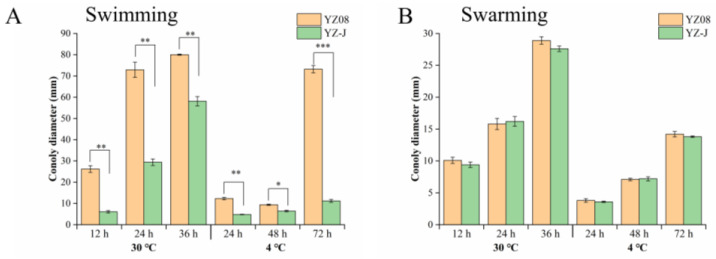
Swimming (**A**) and swarming (**B**) motility of *S. putrefaciens* YZ08 and YZ-J in LB performed at 15 and 30 °C. no mark *p* > 0.05, * *p* < 0.05, ** *p* < 0.01, *** *p* < 0.001.

Cell surface characteristics (chemotactic systems) and motility are critical during biofilm formation [38]. *S. putrefaciens* motility (swimming and swarming) is shown in Figure 6. The swimming motility of both YZ08 and YZ-J increased in a time-dependent manner at the optimal growth temperature (30 °C), but swimming behavior began late at low temperatures (4 °C), especially for strain YZ-J (Figure 6A). The swimming ability of YZ08 strain at the two temperatures was stronger than that of strain YZ-J, which may have been due to the stronger movement ability of the polar flagella of YZ08, as previously described for *Vibrio parahaemolyticus* RIMD2210633. [39]. The swimming abilities of the two *S. putrefaciens* strains were stronger than those of *S. baltica* SB02 and *S. baltica* OS155 [24,40]. Differences in genes encoding chemotaxis proteins and the regulation of some differential key genes such as *flg*M, encoding an important regulatory factor for flagella gene expression; *zom*B, encoding a flagellar motor control protein; and genes encoding PilZ domain proteins, may explain the different swimming phenotypes of the two strains [41,42]. In contrast to their strong swimming abilities, the swarming abilities of *S. putrefaciens* YZ08 and YZ-J were weak, and also showed a time dependence (Figure 6B). At 30 °C, the difference in swarming ability between the two strains was not significant, while at 4 °C the swarming ability of YZ08 was slightly stronger than that of YZ-J. The absence of lateral flagella may explain the weaker swarming ability of *Shewanella* spp. relative to *Vibrio* spp. [39,43]. No genes encoding lateral flagella were found in either strain in this study, which would account for their weak swarming ability.

Table 2 lists most of the motility-associated genes in YZ08 and YZ-J. No differences were found between the two strains for the three gene clusters encoding polar flagellins (A-I, A-II, and A-III). A-I contains structural genes coding for sodium-driven motor rings, loops and hook proteins, and assembly and chaperone proteins, as previously described in *Vibrio* [44]. The A-II gene cluster contains genes encoding regulatory proteins, filaments, basal bodies, switch proteins, and export proteins. The deletion of these genes can lead to the loss of motility in bacteria, as previously described in *Pseudomonas fluorescens* F113 [45]. The third cluster (A-III) contains chemotaxis genes, export genes, and regulatory genes that express late flagellar genes encoding filament proteins, motor proteins, and other flagellar-associated secretory proteins, as previously described [46]. In addition to genes encoding flagellins, those coding for chemotaxis proteins are also critical for bacterial motility [47]. The YZ08 strain contained up to 19 genes encoding chemotaxis proteins compared with only 13 for YZ-J (Table 2), which may explain the markedly greater swimming ability of YZ08 relative to that of YZ-J.

### 3.5. Spoilage-Related Metabolic Pathways

*S. putrefaciens* usually generates spoilage metabolites such as total volatile base nitrogen (TVB-N), TMA, and biogenic amines (BAs) in seafood, leading to a decline in its quality [7]. The spoilage potential of *S. putrefaciens* is associated with sulfur metabolism, BAs metabolism, TMA metabolism, and protease secretion [2].

#### 3.5.1. Biogenic Amines (BAs) Metabolism

**Figure 7:**
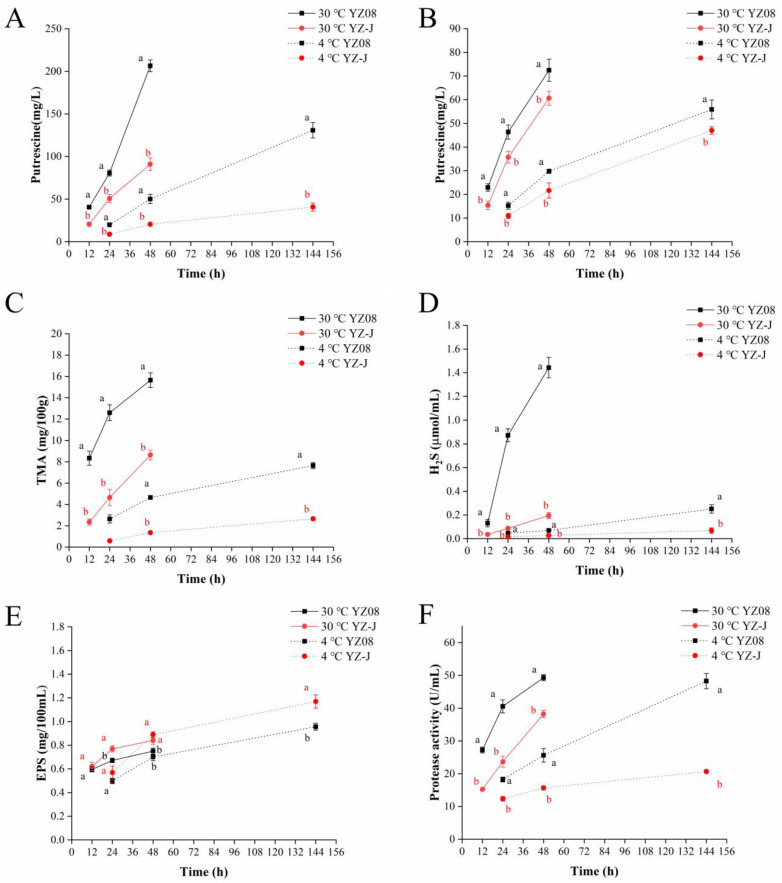
Changes in the putrescine (**A**), cadaverine (**B**), TMA (**C**), H_2_S (**D**), exopolysaccharide (**E**) and protease activity (**F**) produced by *S. putrefaciens* YZ08 and YZ-J during storage at 4 and 30 °C. The significance analysis for different strains at the same culture time and temperature was performed. Different lowercase letters indicate significant differences (*p* < 0.05).

Putrescine, cadaverine, and histamine are common BAs found in spoiled tuna [48]. However, as *S. putrefaciens* is mainly associated with the production of putrescine and cadaverine, and generates only limited amounts of histamine [49]. We focused on investigating the putrescine and cadaverine production activities in the two *S. putrefaciens* strains. The amounts of putrescine and cadaverine produced by YZ08 and YZ-J at 30 and 4 °C using ornithine, arginine, and lysine as substrates are shown in Figure 7A,B. The findings indicated that YZ08 produced greater amounts of putrescine and cadaverine than YZ-J at both the optimum growth temperature and low temperature. We also found that both *S. putrefaciens* strains produced more putrescine than cadaverine, which may be because putrescine can be produced using different substrates (ornithine and arginine) and through different pathways, whereas cadaverine is produced through only one pathway (lysine decarboxylation) [22].

The genomic analysis identified several BA-related genes in the two tested strains (Table 2). Several *pot* genes involved in putrescine metabolism were identified in *S. putrefaciens*, including genes encoding substrate-binding proteins of the putrescine transport system, a putrescine transport ATP-binding protein, spermidine/putrescine ABC transporter permease, putrescine transport system permease protein, and a putrescine-ornithine antiporter. In addition, genes encoding putrescine importer PuuP and gamma-glutamylputrescine oxidoreductase were also found in the genomes of both strains. In *S. putrefaciens* CN32, ornithine decarboxylase is a key enzyme capable of producing putrescine from L-ornithine [2]. Similarly, arginine is converted to cadaverine by ornithine/arginine decarboxylase. The presence of ornithine/arginine decarboxylase corroborated the production of putrescine and cadaverine by the two strains. Although no difference was found in putrescine-related genes, the levels of putrescine and cadaverine production in YZ08 and YZ-J were different, indicating that some regulatory factors could induce the expression of these genes. The results of qRT-PCR supported this hypothesis, indicating that the expression of *spe*C in *S.putrefaciens* YZ08 was significantly higher than that in YZ-J (*p* < 0.01, Figure 5).

#### 3.5.2. TMA Metabolism

As mentioned earlier, most *Shewanella* spp. can reduce TMAO to TMA and produce a fishy odor. Figure 7C shows the amount of TMA produced by YZ08 and YZ-J in LB medium containing TMAO at 4 and 30 °C. At both temperatures, the amount of TMA produced by YZ08 was significantly higher than that of YZ-J (*p* < 0.05). As shown in Table 2, genes encoding trimethylamine N-oxide reductase system protein TorE (K2227_16520), pentaheme c-type cytochrome TorC (K2227_16525), trimethylamine-N-oxide reductase TorA (K2227_16530), molecular chaperone TorD (K2227_16535), histidine kinase TorS (K2227_16540), periplasmic protein TorT (K2227_16545), and response regulator TorR (K2227_16550) were found in YZ08, but not found in YZ-J. The same TMA metabolism related genes were identified in other strains (*S. baltica* OS155 and 128) [13,50]. Although no trimethylamine metabolism-related genes were found in YZ-J, this strain also produced small amounts of TMA, suggestive of the existence of other trimethylamine metabolism pathways. It has been reported that gut microbiota can metabolize compounds containing trimethylamine groups to produce TMA from the precursors of TMA containing choline, phosphatidylcholine, and glycerophosphatidylcholine. The key genes involved in this process are *cut*C, encoding a choline TMA-lyase and gene *cut*D, encoding a choline TMA-lyase activase [51]. In the present study, *pfl*A/D genes, homologs of *cut*C/D were found in *S. putrefaciens* YZ08 and YZ-J. *cut*D and *pfl*D are related to pyruvate formate lyase activating enzyme, and *cut*C and *pfl*A are homologous to pyruvate formate lyase. Therefore, a small amount of TMA produced in *S. putrefaciens* YZ-J may be related to the presence of *pfl*A/D.

#### 3.5.3. Sulfur Metabolism

H_2_S gas has a characteristic off-odor and is associated with the presence of *Shewanella* spp. during the spoilage of seafood [2]. In this study, we explored the H_2_S content produced by *S. putrefaciens* YZ08 and YZ-J (Figure 7D). At 30 °C, YZ08 produced a significant amount of H_2_S in the fish juice. However, at the low temperature (4 °C), both strains generated low amounts of H_2_S at the end of storage (144 h). In general, YZ08 metabolized more H_2_S than YZ-J. The genes associated with sulfur metabolism in YZ08 and YZ-J are listed in Table 2. Sulfate is converted to adenosine 5′-phosphosulfate (APS) by sulfate adenylyltransferase (encoded by the *cys*N gene). APS is then converted to 3′-phosphonoadenosine-5′-phosphate sulfate (PAPS) by the action of adenylyl-sulfate kinase (encoded by *cys*C), which is then further reduced to sulfite by phosphonoadenosine phosphate reductase (encoded by *cys*H). Finally, sulfite is reduced to sulfide by dissimilatory sulfite reductase (encoded by *sir*A). Moreover, the *ttr*SRBC encoding tetrathionate response regulatory protein, tetrathionate sensor histidine kinase, tetrathionate reductase subunit B and cysteine synthase C was also identified in the genomes of both *S. putrefaciens* strains, suggesting that tetrathionate may be reduced and eventually form sulfide through the activity of these enzymes, consistent with the findings of Leustek et al. [52]. That the two strains contained the same sulfur metabolism genes, suggests that they produce different amounts of H_2_S. This could be explained by differences in the transcription levels given that the level of *Sir*A was significantly greater in YZ08 than in YZ-J (*p* < 0.01) (Figure 5). Highly similar genes related to sulfur metabolism were found in *S. baltica* 128 and *S. putrefaciens* YZ07.

#### 3.5.4. Biofilm and Exopolysaccharide Formation

Biofilms have a strong adhesive ability, and they envelop bacteria, thereby enhancing their resistance to adverse environments [53]. On the surface of food processing equipment, some spoilage microorganisms, and pathogenic microorganisms form biofilms. These biofilms are resistant to disinfectants and are difficult to clear, thus affecting food quality and safety. In this study, both strains of *S. putrefaciens* produced biofilms; however, YZ-J produced a significantly greater amount of biofilm than YZ08 at both temperatures (4 and 30 °C) tested (Figure 4B). The genes associated with biofilm formation in YZ08 and YZ-J are listed in Table 2. The key factors regulating biofilm formation of *Escherichia coli* and *Pseudomonas aeruginosa* include c-di-GMP regulatory system, the cAMP/Vfr pathway, and the two-component regulatory system GacS-GacA and EnvZ-ompR [54,55]. The presence of the above genes in the genomes of both YZ08 and YZ-J suggested that there may be multiple pathways regulating biofilm formation in two strains. The mechanisms involved in biofilm regulation in *Shewanella* spp. are poorly understood but are thought to be primarily related to the c-di-GMP pathway. c-di-GMP is synthesized by diguanylate cyclase (DGC) from two molecules of GTP and is decomposed into two molecules of GTP through the activity of phosphodiesterase (PDE) [56]. Several genes encoding DGC and PDE were found in the genomes of both YZ08 and YZ-J (data not shown). However, the *cdg*C gene encoding c-di-GMP PDE was only found in YZ08 (Table 2). Both *Shewanella putrefaciens* CN32 and *Shewanella oneidensis* MR-1 possess a conserved operon containing seven genes [57,58], and this operon also exists in YZ08 and YZ-J. The operon encodes an adhesion protein BpfA; a type I secretion system responsible for the secretion of BpfA into the extracellular compartment (a type I secretion system permease/ATPase, a HlyD family type I secretion periplasmic adaptor subunit, a TolC family outer membrane protein and an OmpA family protein); the protease that regulates BpfA activity (transglutaminase-like cysteine peptidase) and the c-di-GMP receptor protein (EAL domain-containing protein). The secretion of the adhesion protein BpfA in *Shewanella* promotes bacterial adhesion to solid surface, and the bacteria lacking this protein cannot form biofilms [59].

When the intracellular c-di-GMP content is low, the transcription factor FlrA can promote flagellar operon transcription and repress *bpf*A operon transcription by directly binding to the promoter region of *bpf*A, and ultimately biofilm formation is inhibited. When the intracellular c-di-GMP level is high, c-di-GMP binds to and forms a complex with the transcription factor FlrA, thereby relieving the transcriptional activation of flagellar-related genes and the transcriptional repression of the *bpf*A operon. Eventually, the bacterium undergoes irreversible initiation of adsorption and biofilm formation [59]. c-di-GMP also activates the transcriptional regulator RpoS, thereby upregulating the expression of biofilm-associated genes [24]. The amount of biofilm of YZ-J was greater than that of YZ08, which may be due to the higher content of c-di-GMP and the weak motility in YZ-J. Although there are many regulatory mechanisms for biofilm formation, the mechanism for biofilm formation in *Shewanella* spp. mainly involves regulation of the secretion of the adhesion protein BpfA by the FlrA factor. Accordingly, we explored the differences in the expression levels of *flr*A and *bpf*A between the two strains. The RT-qPCR results showed that the expression of *flr*A, encoding an inhibitor of biofilm formation, was significantly higher, and that of *bpf*A significantly lower, in the YZ08 strain than in the YZ-J strain (both *p* < 0.01) (Figure 5), which was in line with the higher amount of biofilm formation in strain YZ-J relative to that in strain YZ08.

Exopolysaccharide is an important component of bacterial biofilms, and bacteria can promote microcolony formation and biofilm maturation by regulating exopolysaccharide synthesis [60]. Similar to the pattern of biofilm formation, the levels of exopolysaccharide produced by YZ-J were significantly higher than those generated by YZ08 at the end of storage (Figure 7E). The genes responsible for exopolysaccharide synthesis in both strains are listed in Table 2. No genes responsible for the biosynthesis of the polysaccharides alginate, Psl, Pel, or that of any other exopolysaccharide, were identified in the genome of either strain. Only glycogen synthesis genes were found. Glucose 6-phosphate is converted to glucose 1-phosphate by the phosphoglucomutase (encoded by *pgm*), following which glucose 1-phosphate is converted to ADP-glucose through the activity of glucose-1-phosphate adenyltransferase (encoded by *glg*C). ADP-glucose is subsequently used to extend the α-1,4-glucosidic chain through glycogen synthase (encoded by *glg*A), after which branching enzyme (encoded by *glg*B) catalyzes the formation of α-1,6-linked branch chains, yielding glycogen. Glycogen is broken down into glucose by glycogen phosphatase (encoded by *glg*P) [61]. In our study, RT-qPCR results (Figure 5) showed that the expression of *glg*A in *S. putrefaciens* YZ-J was significantly higher than in *S. putrefaciens* YZ08 (*p* < 0.001), which could explain the higher production of exopolysaccharides in the former.

#### 3.5.5. Protease and Lipase

**Figure 8:**
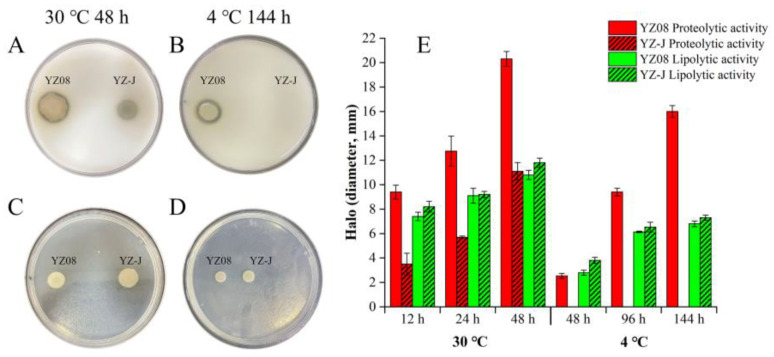
Halos associated with proteolytic (measured on agar plates containing 5% skimmed milk) (**A**,**B**) and lipase (measured on triglyceride agar) (**C**,**D**) activity of *Shewanella putrefaciens* YZ08 and YZ-J following incubation at 4 and 30 °C for 48 and 144 h. Bars in the graphs represent the mean diameter of clear proteolytic and lipolytic halos at 30 °C for 12, 24, and 48 h and 4 °C for 48, 96, and 144 h (**E**).

Proteases and lipases secreted by spoilage bacteria hydrolyze, respectively, protein and fat in seafood, thus reducing its quality [62]. The protease and lipase activity of *S. putrefaciens* YZ08 and YZ-J is shown in Figure 7F and Figure 8. The protease activity of YZ08 was found to be significantly greater than that of YZ-J (*p* < 0.05). The results also showed that YZ08 had substantially larger protease hydrolysis halos than YZ-J, and that no protease hydrolysis halo was seen for YZ-J at 4 °C (Figure 8A,B). However, the lipolytic activity of YZ08 was slightly lower than that of YZ-J, although the difference was not significant (Figure 8E).

Genes encoding protease and lipase from in the YZ08 and YZ-J genomes are listed in Table 3. There were differences in the genes encoding proteases that contain signal peptides between YZ08 and YZ-J. Signal peptides in enzymes are necessary for enzyme secretion [63], and extracellular protease secreted by bacteria generally contains a signal peptide. Here, we found that YZ08 contained two genes encoding M48 family metalloproteases (K2227_09265 and K2227_17060) and one encoding an M4 family metallopeptidase (Hap) while YZ-J had only one gene encoding M48 family metalloprotease (K3G22_08175). YZ08, but not YZ-J, also contained a gene encoding an alkaline serine protease. Moreover, we found that YZ-J lacked protease activity at 4 °C (no halo was produced on skimmed milk-containing), which may be related to absence of any gene encoding an alkaline serine protease in this strain, which usually still exhibited activity over a large temperature range (0–50 °C) [64]. YZ08 and YZ-J shared the same lipase encoding gene, likely explaining why the two strains showed similar lipolytic activity. Genes encoding alkaline serine proteases have also been found in *S. baltica* 128 and *S. putrefaciens* XY07, while the *hap* gene was found in only *S. baltica* 128. Genes encoding lipases were found in both *S. baltica* 128 and *S. putrefaciens* XY07.

## 4. Conclusions

In this study, we analyzed the phenotypic traits (environmental stress, BAs metabolism, TMA metabolism, sulfur metabolism, biofilm formation, exopolysaccharide production, motility, extracellular protease, and lipase activity) and the whole genomes of *S. putrefaciens* YZ08 and YZ-J to identify the genomic determinants of their spoilage-related phenotypes. Although YZ08 and YZ-J were found to be genetically similar, the phenotypic analysis indicated that significant differences in responses to NaCl stress, motility, and spoilage-related metabolism existed between the two strains. Strain YZ08 displayed better growth than YZ-J under NaCl stress, which may be relevant to the presence of more genes encoding sodium:proton antiporter and the high expression of a gene encoding a choline/glycine/proline betaine transporter protein in the YZ08 strain. YZ08 also was found to have greater swimming motility than YZ-J, which was consistent with the greater number of *che*X genes found in the former strain. The strong swimming motility and the low transcript levels of the *bpf*A gene, possibly due to low c-di-GMP content, likely resulted in a low biofilm-forming capacity for YZ08. The lower production of exopolysaccharides in YZ08 relative to YZ-J may be related to the low expression of *glg*A, which encodes glycogen synthase. The lack of the TMA metabolism-related operon *tor*ECADSTR may explain the lower TMA generation in YZ-J. The presence of genes encoding extracellular proteases (alkaline serine protease and M4 family metallopeptidase) may be important factors causing low extracellular protease activity of YZ-J. Overall, some differences in the genetic factors of two strains were consistent with the phenotypic differences. This study contributes to the understanding of the molecular mechanisms underlying the spread, motility, and spoilage activity of two strains of *S. putrefaciens*.

## Figures and Tables

**Table 1:** Stress-related genes of *S. putrefaciens* YZ08 and YZ-J.

Stress	Gene	Encoded Protein	Locus Tag	
			YZ08	YZ-J
Temperature	*grp*E	Heat shock protein GrpE	K2227_15635	K3G22_06000
	*dna*K	Molecular chaperone DnaK	K2227_17110	K3G22_14795
	*dna*J	Molecular chaperone DnaJ	K2227_04710 K2227_09435 K2227_17105	K3G22_07810 K3G22_14790 K3G22_15110
	*hsl*R	Heat shock protein 15	K2227_00795	K3G22_18295
	*hsl*J	Heat shock protein HslJ	K2227_17775	K3G22_03895
	*ibp*A	Heat shock protein IbpA	K2227_12130	K3G22_10395
	*htp*X	Heat shock protein HtpX	K2227_13200	K3G22_11625
	*csp*A	Cold shock protein	K2227_07410 K2227_08825 K2227_12570	K3G22_06460 K3G22_07485 K3G22_10845
	*groe*S	Co-chaperonin GroES (heat shock protein)	K2227_03200	K3G22_02865
	*groe*L	Molecular chaperone GroEL (heat shock protein)	K2227_03205	K3G22_02870
pH	*atp*C/*atp*D/*atp*G/*atp*A/*atp*H/*atp*F/*atp*E/*atp*B	F0F1 ATP synthase	K2227_21885 K2227_21890 K2227_21895 K2227_21900 K2227_21905 K2227_21910 K2227_21915 K2227_21920	K3G22_19335 K3G22_19340 K3G22_19345 K3G22_19350 K3G22_19355 K3G22_19360 K3G22_19365 K3G22_19370
	*pho*A/*pho*D	Alkaline phosphatase	K2227_03285 K2227_03290 K2227_04030	K3G22_15755
NaCl	*nha*D/*nha*C/*Yui*F	Sodium: proton antiporter	K2227_06070 K2227_06375 K2227_10490 K2227_10855 K2227_17710 K2227_20195	K3G22_03965 K3G22_05205 K3G22_09220 K3G22_17770
	*env*Z	Osmolarity sensor histidine kinase EnvZ	K2227_21175	K3G22_00555
	*omp*R	Transcriptional regulatory protein OmpR	K2227_21170	K3G22_00560
		Choline/glycine/proline betaine transport protein	K2227_07790	K3G22_06825
		Plasma membrane protein involved in salt tolerance	K2227_01670	K3G22_01330
Nutrient	*pur*A	Adenylosuccinate synthetase	K2227_03495 K2227_16420	K3G22_03065 K3G22_14060
	*thi*C	Hydroxymethyl-pyrimidine synthase	K2227_11880	K3G22_09485
	*pab*B	Aminodeoxychorismate synthase component 1	K2227_10065	K3G22_08745
	*pan*C	Pantothenate synthetase	K2227_04225	K3G22_15605
	*arg*G	Argininosuccinate synthase	K2227_20520	K3G22_18080
	*cys*M	Cysteine synthase B	K2227_05310	K3G22_04610
	*cys*K	Cysteine synthase A	K2227_13820	K3G22_11825
	*met*H	Methionine synthase	K2227_04845 K2227_16165	K3G22_13880 K3G22_14750
	*gln*A	Glutamine synthetase	K2227_01730 K2227_05875	K3G22_01380 K3G22_05075
	*thr*C	Threonine synthase	K2227_16000	K3G22_13695

**Table 2:** Genes associated with motility, biogenic amine metabolism, trimethylamine metabolism, sulfur metabolism, biofilm formation, and exopolysaccharide formation in *S. putrefaciens* YZ08 and YZ-J.

	Gene	Encoded Protein	Locus Tag	
			YZ08	YZ-J
Motility	A-I	Basal body rod, rings, hook, and regulation protein	K2227_15200-K2227_15275	K3G22_12995-K3G22_13070
	A-II	Filament, basal body, switch, and export proteins	K2227_15080-K2227_15195	K3G22_12875-K3G22_12990
	A-III	Regulatory, export, chemotaxis, and motor proteins	K2227_15020-K2227_15075 K2227_15610 K2227_15615 K2227_03500 K2227_03505	K3G22_12815-K3G22_12870 K3G22_17150 K3G22_17155 K3G22_06020 K3G22_06025
	*che*X	Chemotaxis proteins	K2227_03560 K2227_11490 K2227_11495 K2227_11500 K2227_11510 K2227_11515 K2227_11520 K2227_13440 K2227_14420 K2227_15020 K2227_15025 K2227_15050 K2227_15055 K2227_15255 K2227_15260 K2227_15040 K2227_15045 K2227_16425K2227_19145	K3G22_03130 K3G22_11550 K3G22_12430 K3G22_12815 K3G22_12820 K3G22_12835 K3G22_12840 K3G22_12845 K3G22_12850 K3G22_13050 K3G22_13055 K3G22_14065 K3G22_16555
	*zom*B	Flagellar motor control protein ZomB	K2227_12675	K3G22_10945
	*pil*Z	Pilz domain-containing protein	K2227_08445 K2227_12485 K2227_16555 K2227_16565	K3G22_07390 K3G22_10760 K3G22_14160 K3G22_14170
	*mcp*	Methyl-accepting chemotaxis protein	K2227_01520 K2227_01560 K2227_04495 K2227_05180 K2227_05395 K2227_05730 K2227_05985 K2227_07050 K2227_09095 K2227_09850 K2227_10100 K2227_11990 K2227_13265 K2227_14075 K2227_14105 K2227_15425 K2227_15910 K2227_15945 K2227_17160 K2227_17180 K2227_17310 K2227_17445 K2227_17510 K2227_17895 K2227_18115 K2227_18355 K2227_18980 K2227_20645 K2227_20825 K2227_21180	K3G22_01230 K3G22_01265 K3G22_01935 K3G22_02480 K3G22_02665 K3G22_02720 K3G22_03550 K3G22_04190 K3G22_04340 K3G22_04510 K3G22_04680 K3G22_05125 K3G22_05590 K3G22_06750 K3G22_06810 K3G22_08945 K3G22_09970 K3G22_10280 K3G22_10305 K3G22_12080 K3G22_13645 K3G22_14785 K3G22_15015 K3G22_15195 K3G22_15940 K3G22_16410 K3G22_18525
Biogenic amines metabolism	*puu*P	Putrescine importer PuuP	K2227_02810	K3G22_02305
	*puu*B	Gamma-glutamylputrescine oxidoreductase	K2227_05900	K3G22_05100
	*pot*F	Putrescine transport system substrate-binding protein	K2227_05880	K3G22_05080
	*pot*G	Putrescine transport ATP-binding protein	K2227_05885	K3G22_05085
	*pot*H	Spermidine/putrescine ABC transporter permease	K2227_05890	K3G22_05090
	*pot*I	Putrescine transport system permease protein	K2227_05895	K3G22_05095
	*pot*E	Putrescine-ornithine antiporter	K2227_20335	K3G22_17905
	*spe*C	Ornithine/lysine decarboxylase	K2227_20330	K3G22_17900
	*spe*A	Biosynthetic arginine decarboxylase	K2227_09155	K3G22_07940
	*lys*E	Lysine transporter	K2227_05120 K2227_10525	K3G22_02835 K3G22_06765
Trimethylamine metabolism	*tor*E	Trimethylamine N-oxide reductase system protein TorE	K2227_16520	—
	*tor*C	Pentaheme c-type cytochrome TorC	K2227_16525	—
	*tor*A	Trimethylamine-N-oxide reductase TorA	K2227_16530	—
	*tor*D	Molecular chaperone TorD	K2227_16535	—
	*tor*S	TMAO reductase system sensor histidine kinase/response regulator TorS	K2227_16540	—
	*tor*T	TMAO reductase system periplasmic protein TorT	K2227_16545	—
	*tor*R	Two-component system response regulator TorR	K2227_16550	—
	*pflD*	Formate C-acetyltransferase	K2227_13855	K3G22_11865
	*pfl*A	Pyruvate formate lyase 1-activating protein	K2227_13860	K3G22_11870
Sulfur metabolism	*cys*E	Serine O-acetyltransferase	K2227_12190	K3G22_10455
	*cys*Z	Sulfate transporter	K2227_13805	K3G22_11810
	*cys*K	Cysteine synthase A	K2227_13820	K3G22_11825
	*cys*Q	3′(2′),5′-bisphosphate nucleotidase	K2227_00930 K2227_07435	K3G22_18190 K3G22_06480
	*cys*J	Sulfite reductase [NADPH] flavoprotein alpha-component	K2227_04610	K3G22_15255
	*cys*I	Sulfite reductase (NADPH) hemoprotein, beta-component	K2227_04615	K3G22_15250
	*cys*H	Phosphoadenylyl-sulfate reductase	K2227_04620	K3G22_15245
	*cys*D	Sulfate adenylyltransferase subunit 2	K2227_04650	K3G22_15150
	*cys*N	Sulfate adenylyltransferase subunit 1	K2227_04655	K3G22_15145
	*cys*C	adenylyl-sulfate kinase	K2227_04665	K3G22_15135
	*cys*A	Sulfate transport system ATP-binding protein	K2227_05290	K3G22_04590
	*cys*W	Sulfate transport system permease protein	K2227_05295	K3G22_04595
	*cys*U	sulfate/thiosulfate ABC transporter permease	K2227_05300	K3G22_04600
	*cys*P	Thiosulfate ABC transporter substrate-binding	K2227_05305	K3G22_04605
	*cys*M	Cysteine synthase B	K2227_05310	K3G22_04610
	*sir*A	Dissimilatory sulfite reductase SirA	K2227_19420	K3G22_16750
	*phs*A	Thiosulfate reductase	K2227_02935	K3G22_02440
	*met*B	Cystathionine gamma-synthase	K2227_02960	K3G22_02465
	*sse*A	3-mercaptopyruvate sulfurtransferase	K2227_05850	K3G22_05050
	*met*A	Homoserine O-succinyltransferase	K2227_07535	K3G22_06595
	*ttr*R	Tetrathionate response regulatory protein	K2227_18710	K3G22_02690
	*ttr*S	Tetrathionate sensor histidine kinase	K2227_18715	K3G22_02685
	*ttr*B	Tetrathionate reductase subunit B	K2227_18720	K3G22_02680
	*ttr*C	Tetrathionate reductase subunit C	K2227_18725	K3G22_02675
	*ttr*A	Tetrathionate reductase subunit A	K2227_18730	K3G22_02670
	*glp*E	Thiosulfate sulfurtransferase	K2227_21760	K3G22_00255
Bilofilm formation	*gsp*X	Type II secretion system protein	K2227_00800 K2227_00805 K2227_00810 K2227_00815 K2227_00820 K2227_00825 K2227_00830 K2227_00835 K2227_00840 K2227_00845 K2227_00850 K2227_00855 K2227_06060 K2227_06065	K3G22_02165 K3G22_05200 K3G22_18235 K3G22_18240 K3G22_18245 K3G22_18250 K3G22_18255 K3G22_18260 K3G22_18265 K3G22_18270 K3G22_18275 K3G22_18280 K3G22_18285 K3G22_18290
	*omp*R	Two-component system, OmpR family, phosphate regulon response regulator OmpR	K2227_21170	K3G22_00560
	*env*Z	Two-component system, OmpR family, osmolarity sensor histidine kinase EnvZ	K2227_21175	K3G22_00555
	*cya*A	class I adenylate cyclase	K2227_19915	K3G22_17425
	*csg*B	Minor curlin subunit	K2227_04215	K3G22_15615
	*cpd*A	3′,5′-cyclic-AMP phosphodiesterase	K2227_03910	K3G22_03185
	*crp/vfr*	cAMP-activated global transcriptional regulator CRP	K2227_18785	K3G22_02620
	*trp*E	anthranilate synthase component 1	K2227_13995	K3G22_11985
	*trp*G	aminodeoxychorismate/anthranilate synthase component II	K2227_14000	K3G22_11990
	*msh*E	MSHA biogenesis protein MshE	K2227_02690	K3G22_02185
	*msh*B	MSHA pilin protein MshB	K2227_02705	K3G22_02200
	*msh*A	MSHA pilin protein MshA	K2227_02710	K3G22_02205
	*msh*C	MSHA pilin protein MshC	K2227_02715	K3G22_02210
	*msh*D	MSHA pilin protein MshD	K2227_02720	K3G22_02215
	*flr*C	Two-component system, response regulator FlrC	K2227_15155	K3G22_12950
	*flr*B	Two-component system, sensor histidine kinase FlrB	K2227_15160	K3G22_12955
	*flr*A/*fle*Q	Sigma-54 dependent transcriptional regulator	K2227_15165	K3G22_12960
	*fli*A	RNA polymerase sigma factor FliA	K2227_15060	K3G22_12855
	*flg*M	Flagellar biosynthesis anti-sigma factor FlgM	K2227_15270	K3G22_13065
	*lux*S	S-ribosylhomocysteine lyase	K2227_17240	K3G22_04260
	*csr*A	carbon storage regulator CsrA	K2227_16070	K3G22_13785
	*gac*S/*bar*A	Two-component sensor histidine kinase	K2227_16195	K3G22_13910
	*gac*A/*uvr*Y	Two-component response regulator transcription factor	K2227_13250	K3G22_07880
	*rpo*S	RNA polymerase sigma factor RpoS	K2227_16100	K3G22_13815
	*rpo*N	RNA polymerase factor sigma-54	K2227_03400	K3G22_02925
	*rpo*D	RNA polymerase sigma factor RpoD	K2227_06000	K3G22_05135
	*aph*B	LysR family transcriptional regulator, AphB	K2227_17170	K3G22_04330
	*hfq*	RNA chaperone Hfq	K2227_18885	K3G22_16320
	*fis*	DNA-binding transcriptional regulator Fis	K2227_19855	K3G22_17135
	*crr*	PTS glucose transporter subunit IIA	K2227_12010	K3G22_10300
	*hap*	M4 family metallopeptidase	K2227_02450	-
	*cdg*C	c-di-GMP phosphodiesterase	K2227_03335	-
	*dks*A	RNA polymerase-binding protein DksA	K2227_04250	K3G22_15580
	*gcv*A	Transcriptional regulator GcvA	K2227_15595	K3G22_06040
	*gcv*R	glycine cleavage system transcriptional repressor	K2227_13180	K3G22_08205
	*arc*A	Two-component system response regulator ArcA	K2227_18630	K3G22_02750
	*bpf*A	Biofilm-promoting protein BpfA	K2227_19940	K3G22_17455
		Type I secretion system permease/ATPase	K2227_19945	K3G22_17460
		HlyD family type I secretion periplasmic adaptor subunit	K2227_19950	K3G22_17465
		TolC family outer membrane protein	K2227_19955	K3G22_17470
		OmpA family protein	K2227_19960	K3G22_17475
		Transglutaminase-like cysteine peptidase	K2227_19965	K3G22_17480
		EAL domain-containing protein	K2227_19970	K3G22_17485
Exopolysaccharide formation	*glg*A	Glycogen synthase	K2227_15730	K3G22_05905
	*glg*C	Glucose-1-phosphate adenylyltransferase	K2227_15735	K3G22_05900
	*glg*P	glycogen/starch/alpha-glucan phosphorylase	K2227_15740	K3G22_05895
	*glg*B	1,4-alpha-glucan branching protein GlgB	K2227_15750	K3G22_05885
	*pgm*	Phosphoglucomutase	K2227_10900	K3G22_09265

**Table 3:** Genes encoding proteases and lipases of *S. putrefaciens* YZ08 and YZ-J.

	Gene	Encoded Protein	Locus Tag		Signal Peptide
			YZ08	YZ-J	
Protease	*ctp*A	Carboxyl-terminal protease	K2227_00255	K3G22_19255	No
	*hap*	M4 family metallopeptidase	K2227_02450	-	Yes
	*hsl*U	ATP-dependent protease ATPase subunit HslU	K2227_02475	K3G22_02010	No
	*hsl*V	ATP-dependent protease subunit HslV	K2227_02480	K3G22_02015	No
	*tld*D	Metalloprotease TldD	K2227_02775	K3G22_02275	No
	*pmb*A	Metalloprotease PmbA	K2227_02825	K3G22_02340	No
	*deg*S	Outer membrane-stress sensor serine endopeptidase DegS	K2227_03465	K3G22_03035	No
	*spr*T	SprT family zinc-dependent metalloprotease	K2227_04050	K3G22_15735	No
	*glu*P	Rhomboid family intramembrane serine protease	K2227_04770	K3G22_00250	No
	*res*P	Sigma E protease regulator RseP	K2227_07350	K3G22_06405	No
	*clp*P	ATP-dependent Clp endopeptidase proteolytic subunit ClpP	K2227_08100	K3G22_07070	No
	*clp*X	ATP-dependent protease ATP-binding subunit ClpX	K2227_08105	K3G22_07075	No
	*lon*	Endopeptidase La	K2227_08110	K3G22_07080	No
	*bep*A	M48 family metalloprotease	K2227_09265 K2227_17060	K3G22_08175	Yes
	*clp*A	ATP-dependent Clp protease ATP-binding subunit ClpA	K2227_12560	K3G22_10835	No
	*htp*X	Protease HtpX	K2227_13200	K3G22_11625	No
	*soh*B	Protease SohB	K2227_13950	K3G22_11940	No
	*fst*H	ATP-dependent zinc metalloprotease FtsH	K2227_16705	K3G22_14305	No
		Alkaline serine protease	K2227_16455	—	Yes
	*glg*G	Rhomboid family intramembrane serine protease glgG	K2227_21765	K3G22_04125	No
		Transglutaminase-like cysteine peptidase	K2227_19965	K3G22_17475	Yes
Lipase	*pld*A	Phospholipase A	K2227_04640	K3G22_15160	Yes
		Lipase	K2227_13955	K3G22_11945	Yes
		Patatin-like phospholipase family protein	K2227_14815 K2227_19695	K3G22_12635 K3G22_16980	Yes
	*pho*D	Alkaline phosphatase D family protein	K2227_18295	K3G22_15860	Yes
	*rss*A	Patatin-like phospholipase RssA	K2227_08360	K3G22_07315	No

## Data Availability

Data is contained within the article and Supplementary Materials.

## Supplementary Materials

The following supporting information can be downloaded at: https://www.mdpi.com/article/10.3390/foods11091261/s1, Table S1: quantitative real-time PCR primers used in this study; Table S2: unique genes of *S. putrefaciens* YZ08 by pan-genome analysis between *S. putrefaciens* YZ08 and YZ-J.; Table S3: unique genes of *S. putrefaciens* YZ-J by pan-genome analysis between *S. putrefaciens* YZ08 and YZ-J.
