# Supplementary material for: Genomic Analysis of Two Representative Strains of Shewanella putrefaciens Isolated from Bigeye Tuna: Biofilm and Spoilage-Associated Behavior
Source: Foods. 2022 Apr 27;11(9):1261. doi: 10.3390/foods11091261 (PMC9100107; doi:10.3390/foods11091261)
Supplement: Supplementary file 1 [file foods-11-01261-s001.zip › Table S1.pdf]

Table S1. Quantitative real-time PCR primers used in this study.

Gene name	Forward primer (5′-3′)	Reverse Primer (5′-3′)
16 s rRNA	ACTCCTACGGGAGGCAGCAG	GTATTACCGCGGCTGCTGG
K2227_07790 (*S. putrefaciens* YZ08)	GGTTGGCTTTACATTCTCA	TAATCTTCATCGCCTCTTT
K3G22_06825 (*S. putrefaciens* YZ-J)	AACGAGTATCAACCCACCG	AGCCAACCTGCTTTCATCT
K2227_01670 (*S. putrefaciens* YZ08)	TCTTAGTCATTATTGCGATTC	CCTTGGTCACGACCCAGAG
K3G22_01330 (*S. putrefaciens* YZ-J)	TAGTTATTATTGCGATCCTC	TCCTTAGTAACAACCCACA
*spe*F (*S. putrefaciens* YZ08)	CTGGCTAACTTCTTACGGG	GGCGGATGGTATAGTCTTG
*spe*F (*S. putrefaciens* YZ-J)	TGTAGGTGCGGCTGTTGTC	TTTCCGTAGAAGTCAGTGTTA
*flr*A (*S. putrefaciens* YZ08)	TATGGCGATATTTGACAGG	CAAGAGTTAGTTAGCCGAGT
*flr*A (*S. putrefaciens* YZ-J)	TTCTTTACCCGAACCACTG	CTGCCTGTATTACTGATGACG
*bpf*A (*S. putrefaciens* YZ08)	GGGTTTATTCTCACTCACAGG	GCACCAGCACTACTTACGC
*bpf*A (*S. putrefaciens* YZ-J)	TTTACGATCACAGCCAAAG	TCACGCCAGAACTCAACTA
*sir*A (*S. putrefaciens* YZ08)	TTCGCCACAGTCCAACCCT	CAAGCCGAGATAGCCCAGA
*sir*A (*S. putrefaciens* YZ-J)	TGGGTCACATAGGTCAAGG	AAATCTACGGCAGAGTTCA
*glg*A (*S. putrefaciens* YZ08)	CTTTATCGCTCAGCACTATCC	CCAAGACTCATCCTCGTTT
*glg*A (*S. putrefaciens* YZ-J)	ATCCGAATGCCTTTACCAA	ATCCTTTAAGCCACCAACG
